# Molecular Signatures of Hepatitis C Virus (HCV)-Induced Type II Mixed Cryoglobulinemia (MCII)

**DOI:** 10.3390/v4112924

**Published:** 2012-11-08

**Authors:** Giuseppe Sautto, Nicasio Mancini, Massimo Clementi, Roberto Burioni

**Affiliations:** Microbiology and Virology Unit, “Vita-Salute” San Raffaele University, via Olgettina 58, Milan 20132, Italy; Email: mancini.nicasio@hsr.it (N.M.); clementi.massimo@hsr.it (M.C.); burioni.roberto@hsr.it (R.B.)

**Keywords:** hepatitis C virus (HCV), type II mixed cryoglobulinemia (MCII), B-cell non-Hodgkin lymphoma (B-NHL), viral and host factors

## Abstract

The role of hepatitis C virus (HCV) infection in the induction of type II mixed cryoglobulinemia (MCII) and the possible establishment of related lymphoproliferative disorders, such as B-cell non-Hodgkin lymphoma (B-NHL), is well ascertained. However, the molecular pathways involved and the factors predisposing to the development of these HCV-related extrahepatic complications deserve further consideration and clarification. To date, several host- and virus-related factors have been implicated in the progression to MCII, such as the virus-induced expansion of selected subsets of B-cell clones expressing discrete immunoglobulin variable (IgV) gene subfamilies, the involvement of complement factors and the specific role of some HCV proteins. In this review, we will analyze the host and viral factors taking part in the development of MCII in order to give a general outlook of the molecular mechanisms implicated.

## 1. Introduction

Hepatitis C virus (HCV) infection is a major public health problem with an estimated three to four million people infected each year worldwide and about 170 million carriers [1]. More than 350,000 people die annually from HCV-related liver disease, as current therapies are ineffective in a relevant percentage of cases, and also correlated with several side effects [1]. These estimates are even burdened by the extrahepatic aspects of HCV infection. In particular, about 60% of HCV-infected patients present cold-precipitable (cryoprecipitable) and noncryoprecipitable immune complexes that could be associated with the clinical onset of type II mixed cryoglobulinemia (MCII) [2]. This immune complex-mediated vasculitis is characterized by a primary B-cell clonal proliferation accompanied by the deposition of immune complexes composed of complement factors, mono/oligoclonal IgMs with rheumatoid factor (RF) activity bound to oligo/polyclonal IgGs that, in the case of HCV infection, are mostly directed against HCV proteins [3]. These data support a direct role of HCV in the pathogenesis of this lymphoproliferative disorder, together with the fact that 60%–80% of patients with MCII are infected with HCV and that effective anti-HCV treatment induces significant remissions of MCII [4]. However, not surprisingly, a reduction in MCII symptoms was shown also after anti-B-cell treatment (e.g. rituximab) suggesting a concomitant role of the pathogen and the host in the establishment of this autoimmune disorder [5].

It has been reported by several studies that about 10%–60% of HCV-infected patients presenting cryoglobulins are at risk of contracting symptomatic cryoglobulinemia, clinically characterized by association of purpura, weakness, and arthralgia, possibly complicated by severe renal and neurological involvement [5,6]. In more than 50% of these symptomatic patients, the clinical course is relatively benign with a good prognosis and survival rate [7]. However, it is not clear why some patients develop the above complications, even if several epidemiological risk factors have been identified, such as female gender (female/male ratio of about 2:1), advanced age, other associated autoimmune diseases, longer disease duration, or higher cryocrit levels [8,9,10,11,12]. Moreover, 5–10% of patients with cryoglobulinemic vasculitis will develop B-cell malignancies, especially B-cell non-Hodgkin lymphoma (B-NHL), differently to the general HCV-infected population (0.2%–2.6%) [7,13]. It is actually accepted that HCV persistence contributes to oncogenesis by greatly favoring the biased proliferation of immunoglobulin (Ig)-secreting B-cells clones, which together with genetic and environmental factors may lead to mutational events that cause the onset of a malignant lymphoma [7,14,15,16,17].

**Figure 1:**
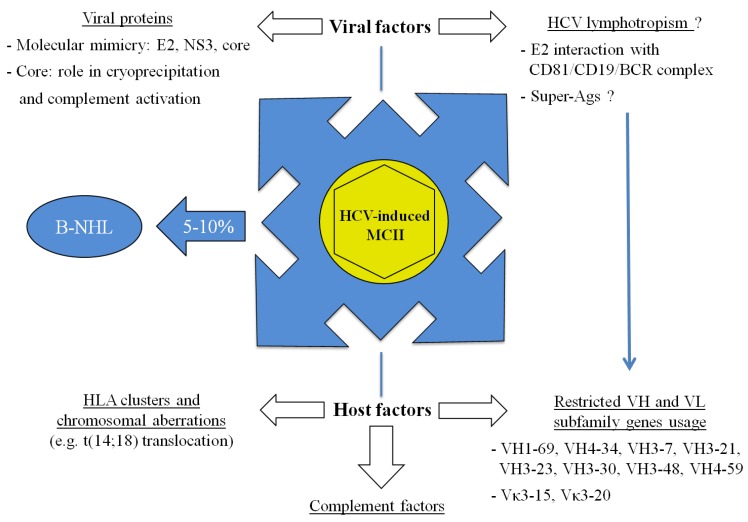
Viral and host molecular factors involved in the development of MCII.

In this review, we will analyze the host and viral factors that have been described to participate in HCV-induced MCII pathogenesis, in order to give an overview of the molecular mechanisms implicated (Figure 1).

## 2. HCV in Induction of MCII

### 2.1. HCV

HCV is an enveloped, positive-stranded RNA virus belonging to the Hepacivirus genus of the *Flaviviridae* family, causing in the majority of cases (about 80%) a chronic infection [18]. On the basis of some conserved regions it can be divided in seven major genotypes and numerous subtypes, differently distributed in the world. In single infected patients, it circulates as a group of highly diversified viral variants, called quasispecies [19].

HCV genome is approximately 9,600 base pairs long and encodes a polyprotein precursor of about 3,000 amino acids. It is cleaved by viral and host proteases, resulting in a series of structural (core, E1 and E2) and nonstructural proteins (p7, NS2, NS3, NS4A, NS4B, NS5A and NS5B) [20]. Virions enter into the host cells, in particular hepatocytes, through a complex and finely regulated multistep process. In brief, the viral envelope type I membrane glycoproteins, E1 and E2 (HCV/E1-E2), allow clathrin-mediated virus endocytosis interacting consecutively with several entry cellular cofactors such as glycosaminoglycans [21,22,23], low-density lipoprotein receptor [24,25], scavenger receptor class B type I [26], the tetraspanin CD81 [27], the tight-junction proteins claudin-1 and occludin, and the recently described Niemann-Pick C1-like 1 cholesterol absorption receptor [28,29,30,31,32]. As expected, the envelope glycoproteins, in particular HCV/E2, are the major targets of the humoral anti-HCV response and, therefore, the most hypervariable HCV proteins [33,34,35]. Recently, increasing data have been evidencing a very complex interplay among different regions of this protein and antibodies (Abs) endowed with highly diverging biological activity, suggesting “novel” mechanisms of HCV escape [36,37,38,39].

### 2.2. HCV Infection and MCII

Every HCV genotype have been found in infection-related MCII, even if different reports describe its higher prevalence among patients infected with HCV of genotype 1 and 2a/c [40,41,42,43,44,45,46]. The reported differences in the prevalence of HCV genotypes in different regions of the world could bias this observation, which should be therefore interpreted with caution.

The mechanisms by which HCV infection leads to RF production, MCII and B-NHL, as well as whether these conditions are related to the lack of some branches of the antiviral immune response are still unknown. The duration of HCV infection required for the development of cryoglobulinemic vasculitis is not well defined but appears to be at least a decade [47]. However, MCII does not display the molecular features of an *in situ* or occult B-cell lymphoma, as evidences show that the B-cell clonal expansion is not a consequence of a true neoplastic process but is probably the result of a pathogenic dysregulation of the host’s immune system. Cryoglobulins are thus the product of virus–host interactions, whose potential pathogenicity derives from several cofactors [48].

As anticipated, in HCV-induced MCII, cryoprecipitates are usually formed by polyclonal IgGs, frequently directed against the HCV core protein and the nucleic acid of HCV, as well as mono/oligoclonal IgM with RF activity [49,50]. Other constituents include also C1q, C-reactive protein (CRP), other HCV antigens (Ags), and molecules of the lectin complement pathway (MBL and MBL-associated serine protease-1), with the latter mostly associated with membranoproliferative glomerulonephritis [51].

Importantly, cryoprecipitation was directly correlated with anticore IgG concentration in the cryoprecipitate, thus inferring that its production is dependent on their selective binding to the Ag in the presence of IgM molecules with RF activity. Indeed, the concentration of HCV RNA in the cryoprecipitate was found to be 10 to 1,000-fold greater than in the supernatant [52,53]. This evidence has suggested a direct role of the HCV core protein in the cryoprecipitation phenomenon [49]. In fact, IgM RF acts as an incomplete cryoglobulin, precipitating at low temperature, probably following a conformational change induced by their binding to IgG with anticore reactivity. In particular, the core is supposed to be the most involved viral protein in cryocrit formation, as demonstrated in the skin and renal tissues of HCV-infected patients with MCII-associated active vasculitis and nephropathy, respectively [54]. In fact, nonenveloped core protein is overproduced during the viral life cycle, and in MCII patients, its plasmatic levels have been associated to cryoglobulinemia-associated symptoms [54]. Moreover, both IgG and IgM may be recognized by the globular heads of C1q interacting with their CH2 and CH3 or CH4 domains, respectively, and for this reason identified as a constituent of cryoprecipitates in some studies. In particular, IgM molecules are good acceptors of C1q and indeed can favor indirect binding of HCV core protein to endothelial cell surface [55,56].

Finally, HCV core protein has also been shown to promote immortalization in different cell lines, as well as being capable of blocking c-myc induced apoptosis and indeed could have a direct role in the pathogenesis of HCV-related lymphomas [57]. At this regard, focusing on animal models, core transgenic mice developed lymphoma with a high frequency (80%) at ages over 20 months [58].

## 3. Molecular Mechanisms Involved in the Establishment of HCV-Related MCII

### 3.1. Molecular Mimicry

The expression of proteins structurally similar to host defense proteins and immunomodulators is an important immune evasion strategy leading to persistence, a mechanism already described for several viruses [59,60]. Moreover, in the case of HCV, this evasion mechanism has been considered also as a possible factor involved in the development of MCII. In particular, some motifs on HCV/E2 glycoprotein have been suggested to be involved in a molecular mimicry of specific Ig portions. More in details, the N-terminal hypervariable region (HVR1) of HCV/E2 shares some conserved motifs with selected human Ig variable (IgV) domains, as well as with the T-cell receptor (TCR) α- and β-chains [61]. This observation, together with the fact that HVR1 acts as an immunodominant “decoy” region diverting the humoral response, would make the frequently elicited anti-HVR1 Abs potentially capable of cross-reacting with other Abs or with TCR [62]. In fact, a lower rate of HVR1 mutations in HCV-positive patients with MCII and presenting a monoclonal IgM expansion with RF activity has been observed [61]. This lower variability in the main target of anti-HCV humoral response was interpreted as a clear sign of impaired immune response, as already observed in agammaglobulinemic or in otherwise immunosuppressed patients [63,64]. Moreover, this low variability at the level of classically hypervariable regions on envelope glycoproteins has been observed also in the case of other viruses, like influenza viruses, suggesting a complex and well-regulated equilibrium between host immune response and viral evasion through variability [65]. Interestingly, a direct correlation has been observed between the degree of similarity of HVR1 to Ig and TCR molecules and the degree of immune escape and persistence in humans and experimentally infected chimpanzees [61]. This indicates that variation in HVR1 sequence is not only correlated with escape from neutralizing Abs, but also to an increased similarity to Ig, suggesting an additional immune evasion strategy through mimicry [66]. As a consequence, this mimicry could determine a chronic stimulation induced also by self-Ags, thus leading to the HCV-related lymphoproliferative disorders.

In addition, a similar mimicry of Ig motifs, not based on identical linear sequences, has been observed between the 1238-1334 amino acid region of HCV/NS3 (in particular in the 1238-1279 and 1251-1270 residues), the viral protease, and the CH3 domain on the Fc portion of human IgG (amino acid residues 345-355) [67]. This region is conserved among all IgG classes except for a single mutation in IgG4. Interestingly, the crystal-structure analysis of a complex between IgM RF and its IgG auto-Ag revealed relatively few Ab contact residues between the potential combining sites of IgM and IgG [68]. In particular, these residues are located on only one side of the combining site surface, indicating that IgM has another, entirely different, specificity than that featured by its RF activity. Autoreactive IgM may thus have originated in response to another Ag, such as HCV/NS3, and the reactivity with IgG Fc may be an unfortunate coincident cross-reactivity phenomenon.

At this regard, certain VH subfamily genes, like the VH4-34, have been described as endowed of binding activity outside the CDRs as clearly evidenced in the case of cold agglutinin-related disease [69,70]. This feature is characteristic of B-cell super-Ags that are supposed to directly activate B cells. As explained more in details below, this could be the case also for the HCV/E2 glycoprotein, due to its ability to stimulate the expansion of a restricted set of VH and VL subfamily expressing B cells [17,71,72]. A similar behavior has also been described for staphylococcal enterotoxins A and D, that function as human B-cell super-Ags rescuing B cell-expressing VH3 and VH4 (including VH4-34) genes inducing cell survival in *in vitro *experiments, and has also been suggested for HIV gp120 [73]. Moreover, certain portions of the FRs seem to be important for super-Ag binding, and thus determinant in the case of a super-Ag-driven selective pressure [70].

Finally, considering the HCV core protein participation in the formation of immune complexes and suppression of T-cell response by interacting with the globular domain of C1q complement receptor (gC1qR), it has been suggested that this interaction may play a key role in determining complement activation, a critical interdependent regulator of the size and solubility of immune aggregates [55,56,74]. In particular, the structural similarity between HCV core Ag and C1q may explain the presence of cross-reactive anti-C1q in HCV-associated MCII [56].

### 3.2. HCV Lymphotropism and Interaction with CD81

Early after its discovery, it was shown that HCV is also a lymphotropic virus, thus suggesting its direct role in lymphoproliferative disorders [75,76]. Interestingly, a lymphotropic isolate of HCV (SB strain) has been obtained from an infected B-cell lymphoma that produces IgM displaying reactivity against the HCV/NS3 protein [77]. Moreover, several studies have described the presence of negative-stranded HCV RNA in B lymphocytes, but these observations were not confirmed in other works [78,79].

HCV/E2 Ag binding to the ubiquitously expressed CD81 receptor on B-cells, as part of the CD81/CD19/CD21 complex, and concomitantly to close anti-HCV/E2 B cell receptors (BCR), could induce a strong B-cell proliferation signal. [80,81]. The binding of HCV/E2 to CD81 induces double-strand DNA breaks and hypermutation of VH (that has been observed also using anti-CD81 Abs) that lower Ab affinity and Ab-mediated complement-dependent cytotoxicity and consequently Ab-mediated neutralization, suggesting a novel escape mechanism of HCV [82]. This process was demonstrated to be dependent on activation-induced cytidine deaminase and related to an increase in the production of TNF-α [83,84]. Moreover, an Ig cloned from B-NHL biopsy specimens have been found to bind HCV/E2 [85].

Indeed, it has been postulated that also anti-HCV IgG complexes with the virus and HCV lipoproteins (VLDL) complexes may act as another B-cell super-Ags inducing the synthesis of non-HCV reactive IgM with RF activity [86]. Then, these auto-Abs, in turn, form immune complexes, which circulate throughout the body and are deposited in small to medium blood vessels, resulting in complement activation and extrahepatic injury.

### 3.3. HCV-Restricted Induction of Determined Ig VH and Vκ Subfamily Genes

Several studies demonstrated the presence, in patients with HCV-related MCII, of IgV gene mutations compatible with a germinal center (GC) or post-GC derivation, a replacement/silent (R/S) mutation ratio consistent with the maintenance of a functional structure of the BCR, and the presence of intraclonal heterogeneity. Conceivably, the similarity in the structure of the variable BCR region and the restricted recruitment of certain IgV gene subfamilies, both for heavy and light chains, may account for selection of B cells expressing specific and similar reactivity. This suggests the possible role of a common Ag, possibly endowed of super-Ag-like features [87,88,89,90,91].

In particular, the WA cross-idiotype Abs, frequently encountered among IgMκ type II mixed cryoglobulins, possess heavy chains belonging to discrete VH subfamily genes, such as VH1-69 and VH3-7 paired with specific Vκ products, such as Vκ3-20 and Vκ3-15, respectively [92]. In fact, it has been observed a major involvement of Vκ expressing B cells, as demonstrated by highly skewed κ/λ ratios and as corroborated by the Vκ usage belonging to these restricted subfamily gene segments. In particular, Vκ3 genes utilize a Jκ1 joining segment for expression, while VH1 genes preferentially use a D3-22 diversity gene segments, with VH1-69 rearranging to JH4 and D region consensus 1, and VH3-7 rearranging to JH3 or JH4 gene segments and D region consensus 2 [93]. Other restricted Ig VH subfamilies have been described in HCV infection and HCV-related lymphoproliferative disorders. Among them the mostly described are the previously mentioned VH4-34 as well as the VH4-59, VH3-7, VH3-21, VH3-23, VH3-30 and VH3-48 [94,95].

VH1-69 is certainly the most recurrent and described Ig VH subfamily induced during anti-HCV infection [96,97,98,99], as well as against other viral pathogens determining acute infections, like influenza viruses [100,101,102,103,104,105,106], and chronic infections, like HIV [107,108]. This VH subfamily gene commonly encodes polyreactive natural Abs and, in HCV-associated cryoglobulinemic patients, it frequently undergoes somatic mutation, probably during affinity maturation. Again, this observation further suggests that HCV-associated MCII and lymphomas may originate in B cells responding to a common Ag. In particular, the VH1-69 gene is highly represented in the anti-HCV/E2 humoral response [71,109]. Preferential use of this gene has also been seen in 10%–20% of patients with CD5+ B cell chronic lymphocytic leukaemia [45,110]. In addition, biased use of VH1-69 has been demonstrated for salivary gland mucosa-associated lymphoid tissue (MALT) lymphomas in which 61% of the patients express this gene [111]. 

Moreover, a significant homology among CDR3 belonging to VH1-69 expanded memory B cells of a patient with HCV-associated MCII and the correspondent region amplified from a monoclonal nonneoplastic B-cell expansion of a Sjögren’s syndrome patient has been described [111]. This suggests that the stimulatory agents underlying these disorders may share common antigenic determinants. Moreover, in the same report, in all the analyzed patients with HCV-associated MCII, 18%–98% of circulating B cells express the VH1-69 gene and, in one-third of these patients, these cells coexpress the Vκ3-20 gene, constituting the previously mentioned WA cross-idiotype [47,71,110,112]. In fact, it has been described that in HCV-infected patients, although the absolute number of circulating B cells was within the normal limits, in some cases, they were almost completely represented by VH1-69 monoclonal B cells and, in patients that had in addition to HCV-associated MCII a splenic lymphoma, a leukemia-like monoclonal expansion of VH1-69 B cells was present [111,113]. This evidence indicates that a clonal population of VH1-69-expressing B cells progressively invades the circulating B cell repertoire of patients with HCV-associated MCII. Moreover, some of these clones have CDR3 sequences identical to RF IgMs isolated from patients with MALT neoplasms [114]. Thus, these nonneoplastic B cells appear to evade the homeostatic mechanisms that regulate the Ag-driven clonal expansion and genetic events may cause further escape from control leading to an absolute lymphocytosis.

In this regard, our group previously reported that the restricted VH1-69 gene usage could be responsible for the B-cell expansion of restricted clones that share the capability of reacting against Igs belonging to this VH subfamily [94]. In particular, it has been observed that the immune repertoire of a patient with HCV-associated MCII contains IgM clones able to react specifically against anti-HCV/E2 Abs belonging to VH1-69 subfamily and derived from the same patient [115]. Indeed, we found that 61% of IgM reactive to anti-HCV/E2 VH1-69 Fab fragments belonged only to two VH subfamilies, VH3-23 (39%) and VH3-21 (22%), that are frequently described in autoimmune disorders [116]. Furthermore, the mutational pattern of selected anti-HCV/E2 IgM showed that almost all clones had a natural origin, evidenced by the high homology to the germline counterpart. Finally, more in details, we found that the VH3-23 subfamily showed a preferential binding of the VH1-69-derived IgG1 Fabs. These data suggest that VH3-23 IgM may be naturally prone to recognize some conserved regions of specific VH subfamilies, as VH1-69, the VH gene described to be elicited in the humoral response against HCV/E2 [35,96,98,99,117]. Considering these data, the HCV/E2-driven stimulation of the immune system may cause the expansion of specific B cells expressing Abs encoded by the VH1-69 subfamily gene and recognized by some natural IgM-encoded subfamily Abs. This could lead to the formation of circulating immune complexes and the cross-linking of BCR by auto-Abs that may allow a chronic activation and a clonal expansion of anti-HCV/E2 B cells. Indeed, a widely held hypothesis is that with increased duration of HCV infection, monoclonal IgM RFs arise from the population of polyclonal IgM RFs that are present before the development of type III cryoglobulins, consisting of polyclonal IgGs and polyclonal IgM RFs, a condition that is believed to precede MCII, characterized by a monoclonal expansion of IgM RF and hypothesized to derive from the population of polyclonal IgM RFs in type III cryoglobulins. However, in this regard, there are conflicting studies [47].

Finally, to reconcile the fact that expansion of B-cell clones carrying the VH1-69 subfamily BCR characterizes the HCV humoral response, as well as that against other viral pathogens, and patient-derived monoclonal IgGs belonging to this subfamily are frequently endowed of a neutralizing activity against their respective pathogens, while IgMs belonging to this subfamily frequently harbor RF characteristics, recent evidences suggest that somatic hypermutation, as well as class switching, abrogate germline BCR reactivity, revealing additional or altered antigenic specificities [118,119,120,121].

In this regard, a pauciclonality of the peripheral memory B-cell population has been described as a unique feature of spontaneous resolving acute HCV-infected patients compared to chronically evolving patients. This finding, considered characteristic only of patients with HCV-associated lymphoproliferative disorders, suggests that the B-cell clones potentially involved in clearance of the virus may also be subjected to abnormal proliferation [122]. However, it is widely accepted that the intrinsic genetic instability of B cells during somatic hypermutation and class-switching processes, may favor genetic aberrations responsible for prolonged B-cell survival and proliferation, thus allowing them to escape from the homeostatic balance controlling clonal expansion. In particular, among chromosomal aberrations, loss of chromosome 2q, gain of the long arm of chromosome 3, 7q deletion and gains of 1q and 8q, have been described [123,124,125]. All these events could lead the lymphoproliferation to become independent of antigenic stimulation, exposing the patients to the risk of developing a frank B-cell malignancy.

Analogously to VH1-69, the VH4-34 subfamily usage has been observed in several autoimmune and lymphorpoliferative disorders, such as diffuse large-cell lymphoma, primary central nervous system lymphoma, B-chronic lymphocytic leukemia, and autoimmune disorders [115]. Moreover, it has also been implicated in HCV response and HCV-associated MCII and lymphomas. Additionally, it is well known that, independently from the DH and JH gene segments, as well as light chains from different subfamilies and isotype associated, the VH4-34 is a naturally autoreactive subfamily [70]. In fact, the VH4-34 gene is found in virtually all cases of cold agglutinin disease in which the red blood cell I/I Ags bind to the FR1 domain of Ig, with a minor involvement of the CDR3 region [69]. Therefore, the high frequency of the VH4-34 gene usage and the intrinsic molecular features of its FR and CDR domains, suggest a possible role of yet unknown B-cell super-Ag in driving HCV-related lymphoproliferative disorders [79].

### 3.4. Complement Factors and Proteins

Low levels of complement components, such as C1, C4 and C2, have been reported in cryoglobulinemic patients, suggesting ongoing complement activation and consumption both via the classical pathway as well as via the MBL pathway. In particular, the C4 level provides a “signature” which may be used to anticipate the presence of significant (> 1 mg/mL) amounts of type II cryoglobulins in the blood [126].

Moreover, in MCII the IgM and IgG deposits are frequently accompanied by the glomerular deposition of MBL, C4, C3 and C1q as a consequence of complement activation at the level of cryoprecipitates, consisting in immune complexes of Ig, Ags of HCV particles and other serum components, such as CRP and complement components [127]. Moreover, possibly present IgMs with RF activity could cover the Fc fragments of IgGs, thus altering their interference with Fc receptors as well as complement activation that is necessary for immune precipitates solubilization with a consequent augmented risk of inflammatory tissue damage. Furthermore, this phenomena could be accentuated with temperature drops or saturation and accumulation of IgM RF-IgG complexes [51].

## 4. B-cell Subsets Involved in MCII

Immunophenotyping and cell-size analysis demonstrated that the expanded B-cell clones induced by HCV infection have a prevalent IgMκ+/CD27+ memory phenotype and, as previously mentioned, with a restricted usage of RF-encoding IgV gene segments in individuals suffering from MCII [113].

As previously seen for the VH1-69-expressing B cells, these expanded B-cell clones frequently replace the entire pool of circulating B cells, although the absolute number of B cells remains within normal limits in the majority of MCII patients, albeit a decrease in naïve B cell population has been observed [128]. This homeostasis of B-cell number is probably maintained by the high rate of B-cell apoptosis induced by HCV, which counterbalances B-cell expansion. However, Racanelli et al., showed that there was a decrease in peripheral blood CD27+ memory B cells in patients with persistent HCV infection and that these cells differentiated into Ig-secreting cells independent of BCR engagement *in vitro *[129]. Moreover, Fournillier and colleagues showed that the levels of naïve and memory B cells and their signaling via BCR stimulation were normal in HCV-infected individuals [130].

Conversely, Roughan *et al.*, reported that some expanded IgM+ CD27+ memory B cells in HCV-infected patients are autoreactive, as evidenced by their functional and genetic characterizations, probably resulting from a lowered immune tolerance due to chronic HCV infection [128]. In fact, Igs secreted from this B-cell subset and isolated from HCV chronically infected patients recognized nuclear Ags and presented long CDRH3 with a net positive charge, typical of autoreactive Abs, and these features were not observed in healthy controls or in infected patients without IgM memory B-cell expansion. Moreover, these expanded B cells have undergone somatic hypermutation concentrated at the level of CDRs, absence of isotype switching and a R/S ratio suggesting of an Ag-driven memory expansion of the IgM subset in the HCV-infected individuals. Finally, the authors observed an increase in ALT or viral load in this group of patients, thus suggesting a possible pathogenetic role concerning the expansion of this B-cell subset [128].

In addition, a polyclonal activation and expansion of CD5+ B cells has been observed during interaction between HCV and lymphocytes and as being associated with HCV infection and HCV-related MCII [45]. The circulating innate CD5+ cells are believed to be equivalent to murine B-1 cells, which have restricted BCR gene segment usage and are primary source of auto-Abs (IgM). However, in this regard, there are conflicting data, as other groups reported that no correlation was found between the increase of CD5+ B cells and the presence of cryoprecipitates or RF in patients with HCV, as well as in those with a HCV-related lymphoproliferative disease [95,113,131].

Finally, as a possible consequence of HCV/E2 binding to CD81, an upregulation of this tetraspanin in HCV-infected patients, with a further upregulation in patients with MCII, has been observed. However, conflicting results have been published showing CD81 downregulation, whereas the cognate-complex CD19 molecule was upregulated on peripheral B lymphocytes in HCV-infected patients with MCII or B-NHL [83,84].

## 5. Conclusions

Several chronic infections are involved in the development of systemic autoimmune-related diseases. It is now accepted that HCV interaction with B cells plays a critical role in providing valid explanations for the occurrence of autoimmune disorders in the course of chronic HCV infection, such as MCII. The state of the actual knowledge in this field has been reviewed in this paper. However, many dark areas are left in the comprehension of several aspects of their pathogenetic mechanisms.

Firstly, the process of B-cell clonal expansion occurring in an environment favorable to the immortalization of one or few specific clones, and the predisposing factors leading to neoplastic transformation, should be clarified. Moreover, as HCV is not a genuine lymphotropic virus, the factors allowing HCV entry and replication in lymphoid cells should be better identified.

Secondly, other factors implicated in the formation and maintenance of a pro-inflammatory and autoreactive environment, responsible of extrahepatic manifestations, like MCII, and to the subsequent development of malignancies need deeper investigations. In this regard, further considerations deserve the mechanisms of the interaction of HCV Ags with BCRs, as well as the subsequent Ag-mediated signaling that may occur, such as: (i) prolonged antigenic stimuli derived from HCV viral proteins that lead to BCR rearrangements and expansion of B-cell clones expressing a restricted set of VH and VL subfamily genes, in particular of those encoding natural Abs with RF activity; (ii) possible mechanisms of BCR revision, together with the specific production of cellular cofactors promoting B-cell proliferation and antiapoptotic signals (like certain overexpressed chemokines and cytokines as Fas, BLyS, BCA-1 and BAFF) or the occurrence of their possible related polymorphisms [83,132,133,134]; (iii) additional events like genomic translocations as those involving the bcl-2 and myc genes (*e.g.*, t(14;18) translocation) [132]. Interestingly, considering the host genetic background, certain HLA combinations have been found to be more prevalent in MCII HCV-infected patients, suggesting they may be associated with increased susceptibility to MCII and that B-cell proliferation could be the consequence of both IgM stimulation and HLA presentation. In this regard, there are some studies reporting an association between the presence of DR5 supertype of HLA-class II DRB1 and DQ3 supertype HLA-class II DQB1 and a significantly increased risk of developing MCII in patients with chronic HCV infection, while other groups reported an association with the prevalence of HLA-class II DR8 and DR1 phenotypes [135,136]. On the contrary, other phenotypes, like HLA-class II DR7, seem to protect against the development of this HCV-related disease [136]. Thirdly, polymorphisms of proteins that tightly regulate the complement system might be deeply investigated in order to determine the environmental and genetic determinants of complement abnormalities characteristics for MCII.

Finally, the wide spectrum of lymphomas that have been described in patients with HCV infection, ranging from lymphoplasmacytoid, to MALT-type, to follicle-center cell lymphomas, seems to indicate that more heterogeneous and complex processes are probably involved in the HCV-associated lymphomagenesis [137,138].
